# Obstructive sleep apnea may increase the risk of Alzheimer's disease

**DOI:** 10.1371/journal.pone.0221255

**Published:** 2019-09-05

**Authors:** Sylwia Przybylska-Kuć, Maciej Zakrzewski, Andrzej Dybała, Paweł Kiciński, Grzegorz Dzida, Wojciech Myśliński, Andrzej Prystupa, Barbara Mosiewicz-Madejska, Jerzy Mosiewicz

**Affiliations:** 1 Chair and Department of Internal Diseases, Medical University of Lublin, Lublin, Poland; 2 1st Military Hospital in Lublin, Department of Internal Medicine, Lublin, Poland; 3 Department of Experimental Hematooncology, Medical University of Lublin, Lublin, Poland; 4 Chair and Department of Internal Diseases, Students Scientific Association, Medical University of Lublin, Lublin, Poland; University of Rome Tor Vergata, ITALY

## Abstract

**Objectives:**

Amyloid-β 1–40 (Aβ 1–40) and amyloid-β 1–42 (Aβ 1–42) are the proteins known to be involved in the pathogenesis of Alzheimer’s disease (AD)–the most common cause of dementia in the elderly. Hypoxia is suspected to be one of conditions associated with Aβ plasma level increase. A common reason of hypoxia is obstructive sleep apnea (OSA), characterized by recurrent episodes of apnea.

**Aim:**

The aim of the study was to evaluate plasma Aβ 1–40 and Aβ 1–42 concentrations in patients with OSA.

**Methods:**

Patients with suspected OSA (n = 112) underwent polygraphic examinations Patients with confirmed OSA (n = 81) showed apnea/hypopnea index greater than or equal to 5. Mild and moderate form of the disease was defined when AHI was 5–30 (n = 38, OSA+), severe–when AHI was >30 (n = 43, OSA++). Individuals with AHI<5 (n = 31) served as control group (OSA-).

**Results:**

Aβ 1–40 concentrations in OSA++ (191.1 pg/ml) group was significantly (p<0.05) higher compared with OSA- (76.9 pg/ml) and OSA+ (159.4 pg/ml) and correlated with selected parameters of hypoxemia severity. There were no differences in Aβ 1–42 concentration between the groups.

**Conclusion:**

In patients with severe OSA Aβ 1–40 plasma concentrations are significantly higher compared with OSA- and OSA+ and seem to be related to hypoxia severity, which may indicate increased risk of AD development in this group of patients.

## Introduction

Alzheimer's disease (AD) is a degenerative disorder of the nervous system of unknown etiology and unclear pathogenesis. It is the most frequent cause of dementia in the elderly [1,2]–there are about 30 million people affected by it around the world [3]. In AD pathogenesis there is deposition of amyloid β (Aβ) in form of senile plaques in extracellular space in the brain and of tau protein in form of neurofibrillary tangles inside the neurons, mainly in the cerebral cortex. Aβ is a polypeptide with a molecular weight of about 4.2 kDa. It contains 39 to 42–43 amino acids and it is synthesized in the brain as a result of amyloidogenic metabolic pathway activation. This leads to cognitive disorders such as impairment of memory, speech and abstract thinking. Cognitive functions disorders, recent memory dysfunction and impairment of planning capability also affect patients suffering from obstructive sleep apnea (OSA). In this condition there are episodes of upper respiratory tract airways closing (apneas) or their narrowing (shallow breathing) while respiratory muscles activity is maintained. This leads to worse blood oxygenation, sympathetic nervous system activation, blood pressure increase, sleep fragmentation and excessive daytime sleepiness. Hypoxia is one of the factors increasing Aβ synthesis. A significant hypoxia-induced increase of Aβ level was observed in animal models [4]. As the episodes of hypoxia also occur in patients with OSA, Aβ synthesis may be increased in this group of patients.

## Material and methods

The study included 112 OSA-suspected Caucasian patients (85 men and 27 women). Mean patients age was 51. Medical history, apnea symptoms, coexisting disorders and Epworth Sleepiness Scale (ESS), was taken from all patients. Obesity was diagnosed when Body Mass Index (BMI) was equal or higher than 30 kg/m^2^. The exclusion criteria were: the presence of apparent neurological, psychiatric, brain vessels diseases, respiratory failure, chronic obstructive pulmonary disease, stages 3–5 of chronic kidney disease, acute infection, thyroid disorders (except from effectively pharmacologically treated hypothyroidism), acute cardiovascular disorders (cardiac arrhythmias, aggravation of chronic heart failure) and sleep-related respiratory disorders other than OSA. Every patient underwent polygraphic examination (PSG) performed from 10 p.m. to 5 a.m. in the Department of Internal Diseases using SleepDoc Porti 8 (Dr. Fenyves und Gut Deutschland GmbH, Germany).Episode of apnea was diagnosed when breathing amplitude decreased by more than 90% for at least 10 seconds. Hypopnea diagnosis criterion was breathing amplitude decrease by at least 30% for at least 10 seconds with concomitant decrease of blood saturation (SaO_2_) by at least 4%. PSG examination parameters subjected to our analysis were as following: number of apnea and hypopnea episodes per hour (apnea/hypopnea index—AHI), number of apnea episodes per hour (apnea index—AI), number of hypopnea episodes per hour (hypopnea index–HI), total hypopnea and apnea duration during the examination (respiratory disturbance time–RDT), hypopnea and apnea duration time per hour (respiratory disturbance time index–RDTI), minimal and mean saturation (SpO_2_), number of desaturation (SaO_2_<90%) episodes per hour (oxygen desaturation index–ODI), percentage of time with saturation lower than 90% (t90), total duration of examination with blood oxygenation lower than 90% (oxygen desaturation time–ODT), duration of desaturation below 90% during one hour of examination (oxygen desaturation time index–ODTI), mean heart rate (HR) during examination.

The studied group consisted of patients with sleep-related breathing disorders classified as obstructive sleep apnea in adults in International Classification of Sleep Disorders (ICSD-3). OSA was diagnosed when AHI was higher or equal to 5 and there were coexisting clinical symptoms. Sleep apnea severity was assessed according to recommendations of the American Academy of Sleep Medicine AASM[5]. Patients with mild or moderate stage (AHI 5–30) belonged to the subgroup called OSA+, while patients with severe stage (AHI>30) were assigned to the subgroup OSA++. The control group (OSA-) consisted of patients with AHI<5 (n = 31).

The blood samples were taken once during the study between 6 and 7 a.m. After centrifugation samples were frozen in -80°C. Aβ 1–40 and Aβ 1–42 levels measurement was performed using immunoenzyme tests (ELISA Kit for Amyloid Beta Peptide 1–40 and ELISA Kit for Amyloid Beta Peptide 1–42, Cloud-Clone Corp., USA).

The study was approved by Bioethics Committee in Medical University of Lublin (KE-0254/156). Written informed consent was obtained from every examined patient.

Statistical analysis was carried out using Statistica 12 (StatSoft, USA) computer programme. Continuous variables were described using arithmetic means along with standard deviations. Convergence of variables distribution with normal distribution assessment was made with Shapiro-Wilk test. Equality of variances was assessed using Levene's test. To determine differences significance there was Student's t-test or Mann-Whitney U test used depending on normality test result. Differences between three groups of patients were assessed with analysis of variance (ANOVA) or its nonparametric equivalent–Kruskal-Wallis test and post hoc comparisons: Scheffe or Dunn's test. Multifactor analysis of variance was used to explore the influence of two or more grouping variables. Influence of accompanying continuous variables on dependent variable was established using analysis of covariance (ANCOVA). Depending on variables distribution Pearson's correlation coefficient or Spearman's rank correlation coefficient was used in order to assess direction and strength of variables association. Qualitative variables were defined with structural indicators. To establish differences significance Chi-Square Test of Independence was used. For cross-tabulation Yates' correction or Fisher exact test was used. Statistical significance <0.05 was employed.

## Results

Based on performed PSG examination there were two groups separated: patients (n = 81) and controls (n = 31). There were 38 patients in OSA+ subgroup and 43 patients in OSA++ subgroup.

General characteristics of the studied groups are shown in Table 1. Patients in the studied group presented following features: older age, predominance of males and higherBMI. Diabetes and hypertension were also more frequent in OSA-positive group.There were no differences between the studied groups in terms of occurrence of obesity, coronary artery disease and chronic heart failure.

**Table 1:** General characteristics of the study groups (OSA+, OSA++) and the controls.

	OSA- (n = 31)	OSA+ (n = 38)	OSA++ (n = 43)	p
Age (years)	46.1±14.1	52.2±10.1	54.4±10.4	<0.05
Men prevalence (%/n)	54.8%/17	81.6%/31	86%/37	<0.05
Women prevalence (%/n)	45.2%/14	18.4%/7	14%/6	<0.05
Height (cm)	171.2±10.1	174±7.7	172±8.8	NS
Body mass (kg)	92.6±21.1	96.7±18.5	105.1±22.7	<0.05
BMI (kg/m^2^)	31.4±6.1	32.1±6.8	35±6.3	<0.05
BSA (m^2^)	2.1±0.3	2.1±0.2	2.2±0.2	NS
ESS (points)	8.5±5.6	10±5.5	12.3±5.9	<0.05
Obesity %/n	64.5%/20	60.5%/23	79.1%/34	NS
Diabetes %/n	6.4%/2	26.3%/10	23.3%/10	0.03
Hypertension %/n	51.6%16	57.9%/22	79.1%/34	<0.05
Coronary artery disease %/n	0	5.3%/2	14%/6	NS
Chronic heart failure %/n	0	2.6%/1	4.6%/2	NS

BMI—body mass index, BSA—body surface area, ESS—Epworth Sleepiness Scale, NS–non significant

As it was expected, in OSA+ and OSA++ groups values of AHI, AI, HI, RDT, RDTI, ODI, ODT, ODTI were higher, while mean and minimal SpO_2_ were lower in comparison with control group. PSG results in particular groups are presented in Table 2.

**Table 2:** Results of simplified night polygraphy in particular groups of patients (abbreviations explanations in the “Materials and methods” section).

	OSA- (n = 31)	OSA+ (n = 38)	OSA++ (n = 43)	p
AHI (h^-1^)	2±1.7	15.7±7.2	54.1±17.7	<0.05
AI (h^-1^)	0.5±0.7	9.2±6.2	41.5±20.1	<0.05
HI (h^-1^)	1.5±1.4	6.7±3.5	12±9.7	<0.05
RDT (min)	6.3±9.6	40.2±28.5	136.4±61.2	<0.05
RDTI (min/h)	1.5±4.1	6.2±4.82	23.8±9.3	<0.05
Mean SpO_2_ (%)	92.6±1.8	92.2±1.9	88.9±3.9	<0.05
Minimal SpO_2_ (%)	85.7±4.3	79.1±7.2	65.3±14.5	<0.05
t90 (%)	3.7±10.2	6.5±13.3	29±22.5	<0.05
ODI (h^-1^)	5.3±6.1	20.2±14.5	59.5±20.7	<0.05
ODT (min)	12.6±11.9	37.1±22.5	128.8±4.3	<0.05
ODTI (min/h)	1.8±1.7	6.4±3.7	25.5±7.1	<0.05
HR (min^-1^)	63±6.4	62.5±9.8	62.3±9.3	NS

NS–non significant

**Fig 1:**
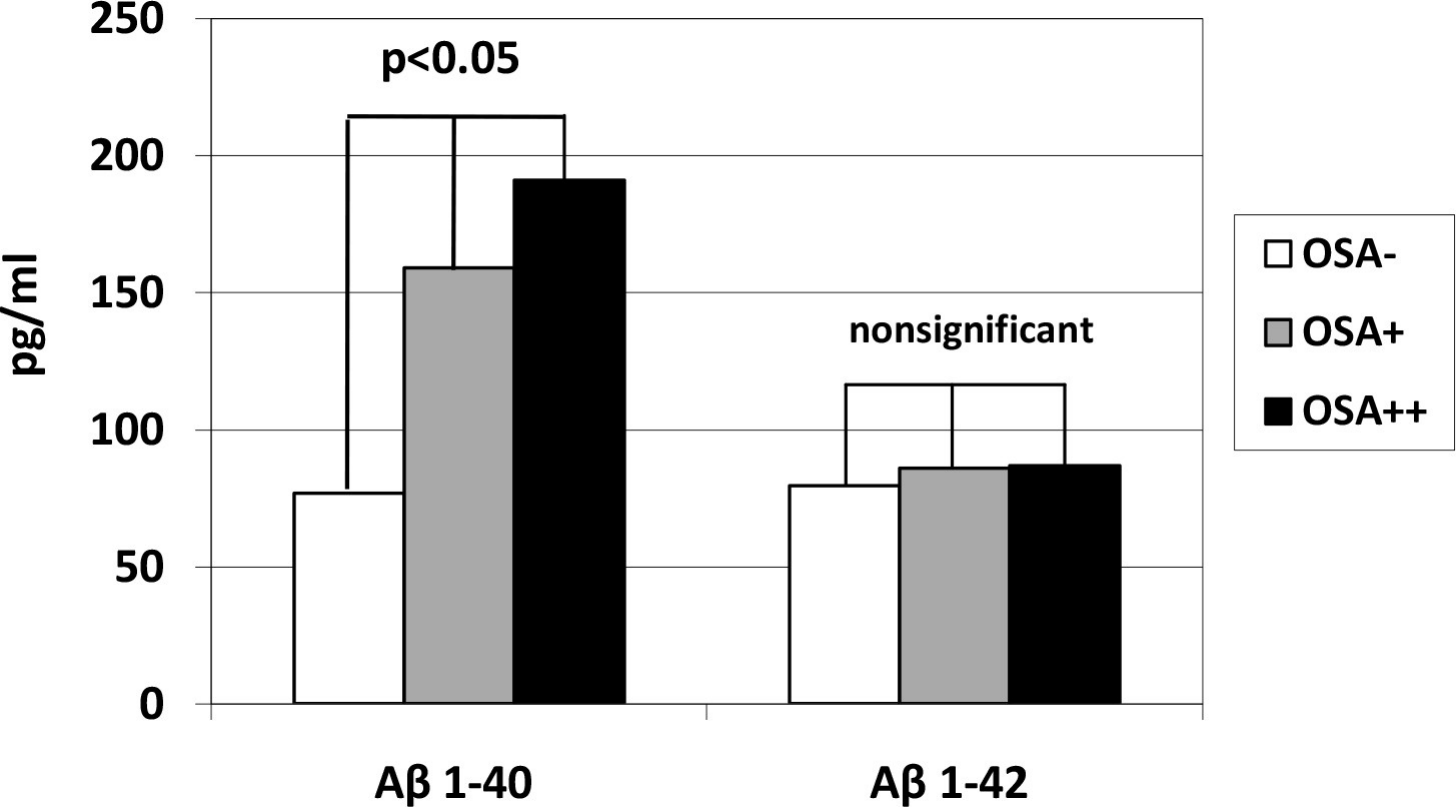
Amyloid β 1–40 (Aβ 1–40) and β 1–42 (Aβ 1–42) plasma levels in Obstructive Sleep Apnea (OSA+, OSA++) and control (OSA-) groups.

Aβ 1–40 concentrations in OSA++ (191.1 pg/ml) group was significantly (p<0.05) higher compared with OSA- (76.9 pg/ml) and OSA+ (159.4 pg/ml). Analysis of covariance and multifactor analysis of variance showed no influence of age, sex, obesity, diabetes, hypertension, chronic heart failure on serum level of Aβ 1–40. There was no significant difference in Aβ 1–42 serum level between OSA+ (85.9±60.1 pg/ml), OSA++ (86.9±53.3 pg/ml) subgroups and control group (79.5±54.1 pg/ml). Aβ 1–40 and Aβ 1–42 serum level results are presented in Fig 1.

Aβ 1–40 serum level was positively correlated with AHI, AI, RDT, RDTI, t90, ODI and negatively correlated with minimal SaO_2_. There was no correlation found between Aβ 1–42 serum level and PSG parameters. Correlation between Aβ 1–40 serum level and PSG parameters are presented in Table 3.

**Table 3:** Spearman’s rank correlation coefficient between amyloid beta 1–40 (Aβ 1–40) and polygraphic parameters (abbreviations explanations in the “Methods” section).

Pair of variables		R Spearman coefficient	p
Aβ 1–40	AHI (h^-1^)	0.45	<0.05
Aβ 1–40	AI (h^-1^)	0.43	<0.05
Aβ 1–40	HI (h^-1^)	0.28	<0.05
Aβ 1–40	RDT (min)	0.48	<0.05
Aβ 1–40	RDTI (min/h)	0.49	<0.05
Aβ 1–40	Mean SpO_2_ (%)	-0.28	<0.05
Aβ 1–40	Minimal SpO_2_ (%)	-0.47	<0.05
Aβ 1–40	t90 (%)	0.41	<0.05
Aβ 1–40	ODI (h^-1^)	0.46	<0.05

## Discussion

Aβ is a product of pro-protein proteolysis–amyloid precursor protein (APP). It is catabolised in two metabolic pathways: physiological–non-amyloidogenic and pathological–amyloidogenic. In the first pathway APP degradation with α and γ secretases leads to production of non-toxic peptide Aβ 17–40 or Aβ 17–42. This takes place in normal ageing process of the brain. In the second (amyloidogenic) pathway secretases β and γ take part in production of toxic Aβ 1–40 and Aβ 1–42 proteins as it was demonstated in Alzheimer's disease. Many research data suggest that higher blood level of Aβ 1–40 and Aβ 1–42 increases the risk of Alzheimer's disease [6,7]. Hypoxia is one of the factors influencing on the increase of toxic Aβ 1–40 and Aβ 1–42 level. Correlation between Aβ level and hypoxia were documented in vitro and in animal models [4,8]. Hypoxia may have a wide-ranging influence on toxic Aβ 1–40 and Aβ 1–42 level. In the experimental studies it was proven that chronic hypoxia in mice caused fast increase of hypoxia inducible factor (HIF-1 α), which increases beta-secretase for beta-site APP cleaving enzyme (*BACE-1*) gene expression. This gene is responsible for increase of enzymatic activity of β secretase. As a result amyloidogenic pathway is activated and higher amount of toxic amyloid is produced [4,8]. Genetic knockout of *BACE-1* gene in mice leads to reduction of β secretase activity and decrease of amyloid production. This confirms the role of *BACE-1* overexpression in increased production of toxic amyloid [9]. In another study 50% hypoxia decreased ADAM-10 protein expression in human neuroblastoma tissue culture. As ADAM-10 protein influences α secretase activity, it may lead to amyloidogenic pathway activation [10]. It has also been observed that in tissue culture hypoxia decreases neprilysin (NEP) zinc metalloprotease activity. It is one of the main amyloid degrading enzymes [11]. These data suggest that hypoxia may lead to overproduction of Aβ and its reduced degradation in the brain. However, correlation between Aβ level and hypoxia in humans has not been clearly documented [12]. Regarding this ambiguity, assessment of Aβ level in OSA, a frequent cause of hypoxia, seems to be an interesting issue. Episodes of upper respiratory tract airways obstruction lead to hypoxia, which is documented with polygraphic examination. Testing Aβ 1–40 and 1–42 serum level and referring them to polygraphy results in our study led us to conclusion that there is a significant correlation between Aβ 1–40 serum level and hypoxia during sleep. What is more, this correlation seems to be quantitative–the more intense hypoxia, the higher Aβ 1–40 serum level. Aβ 1–40 level is not only significantly higher in group with the most exacerbated breathing disorders during sleep, but it is also positively correlated with AHI (r = 0.45; p<0.05) and negatively correlated with SpO_2_ (r = -0.47; p<0.05). Regarding Aβ 1–42 no similar correlations have been found.

Previous studies results are not equivocal. Sun et al. showed increase of Aβ 1–40 level as a result of hypoxia by exploring *BACE-1* gene expression in animal model. This gene is responsible for β secretase activity increase [4]. This process results in amyloidogenic pathway activation and production of higher amount of toxic Aβ. Xian Lee et al. observed increased blood levels of both Aβ 1–40 and Aβ 1–42 in patients with OSA [12]. What is more, in their studies Aβ 1–40 level was positively correlated with OSA and hypoxia intensity, what is similar to our findings. Similarly, Bu et al. showed significantly higher Aβ 1–40 and Aβ 1–42 levels in group of 45 OSA-positive patients when compared to control subjects [13]. In this study Aβ 1–40 blood level was significantly correlated with AHI, desaturation index, mean and minimal SaO_2_ value, similarily to the previously mentioned paper and to our research results as well. According to the authors of the paper mentioned above, analogical correlations applied to Aβ 1–42, which was not observed in our study. Aβ level in hypoxia was also tested in the cerebrospinal fluid (CSF). In a two-year prospective study Sharma et al. observed that Aβ 1–42 level in CSF increases along with OSA intensity [14]. Aβ active transport from CSF to blood was documented in animal models and humans [15,16]. Increased Aβ blood level corresponds with its higher level in CSF [12]. It is worth mentioning that Aβ in blood may also come from peripheral tissues [12].

However, Shiota et al. published different results. In their paper, chronic hypoxia did not correlate with increase of intracellular Aβ 1–40 level in mice's brains. Nevertheless increase of Aβ 1–42 level was observed in these circumstances [8].

According to another paper, decrease of amyloid β 1–42 level is related to increased risk of AD. Researchers found that amyloid β 1–42 level is significantly lower in OSA-positive patients than in control group and treatment with continuous positive air pressure (CPAP) leads to its normalisation [17]. Correlation between amyloid β 1–40 level in CSF and OSA was also explored by Ju et al. Researchers assumed that its higher level was AD risk factor. Unexpectedly, this amyloid fraction level in CSF was lower in OSA-positive patients than in control group. Possible explanation of this fact was that intracranial and intrathoracic pressure fluctuations in OSA patients impair the interaction between CSF in the glymphatic system and interstitial fluid (ISF). Hence, Aβ and other neuronal metabolites tend to accumulate in ISF rather than being transported to CSF. This hypothesis elucidates the phenomenon of diminished Aβ CSF level in OSA and AD patients [18]. These findings are in accordance with research conducted by Bubu et al. [19], who also proved Aβ 1–42 level in CSF to be lower in patients with OSA than in controls. Another study indicating on lower CSF amyloid levels in OSA and AD patients is research by Liguori et al. [20]. In this publication CSF amyloid levels in AD patients were found to be lower than in OSA patients. However, OSA patients presented lower CSF amyloid levels than controls. What is more, the authors found a correlation between AHI and Aβ 1-42/Aβ 1–40 ratio in OSA patients.

The authors of this paper are aware of its limitations. This research needs to be expanded on bigger groups of patients. What is more, amyloid levels were tested in blood, which may not precisely reflect amyloid level in the brain. Nevertheless, CSF amyloid level also does not perfectly mirror its accumulation in ISF of the brain. However, the blood-brain barrier was proven to be damaged in patients suffering from OSA, which allows us to have a better insight into amyloid level in the brain. In addition, some authors suggest that AD is not only disease of the brain itself, but other peripheral organs also take part in its pathogenesis. Hence, amyloid level measurement in blood may shed a light on AD pathogenesis and its relation to OSA and may allow to diagnose early AD stages in the future [21].

## Conclusion

β-amyloid 1–40 serum levels are increased in patients with severe obstructive sleep apnea. This may lead to the incerased risk of Alzheimer's disease.
